# Antifouling Mussel-Inspired Hydrogel with Furanone-Loaded ZIF-8 for Quorum Sensing-Mediated Marine Antifouling

**DOI:** 10.3390/gels11060466

**Published:** 2025-06-18

**Authors:** Yanbin Xiong, Junnan Cui, Xiaodan Liu, Haobo Shu, Pan Cao

**Affiliations:** 1 Shenzhen Institutes of Advanced Technology, Chinese Academy of Sciences, Shenzhen 518055, China; yb.xiong@siat.ac.cn; 2 University of Chinese Academy of Sciences, Beijing 101408, China; 3 School of Mechanical Engineering, Yangzhou University, Yangzhou 225009, China; 4 Jiangsu Key Laboratory of Surface Strengthening and Functional Manufacturing, Yangzhou University, Yangzhou 225009, China; 5 College of Animal Science and Technology, Yangzhou University, Yangzhou 225009, China; 6 Department of Polymer Materials, School of Materials Science and Engineering, Shanghai University, Shanghai 200444, China

**Keywords:** marine biofouling, mussel-inspired hydrogel, ZIF-8, quorum sensing inhibitor, antifouling coating

## Abstract

Marine biofouling, the process of marine microorganisms, algae, and invertebrates attaching to and forming biofilms on ship hulls, underwater infrastructure, and marine equipment in ocean environments, severely impacts shipping and underwater operations by increasing fuel consumption, maintenance costs, and corrosion risks, and by threatening marine ecosystem stability via invasive species transport. This study reports the development of a hydrogel-metal-organic framework (MOF)-quorum sensing inhibitor (QSI) antifouling coating on 304 stainless steel (SS) substrates. Inspired by mussel adhesion, a hydrophilic bionic hydrogel was first constructed via metal ion coordination. The traditional metal ion source was replaced with a zeolitic imidazolate framework-8 (ZIF-8) loaded with 2-(5H)-furanone (HF, a QSI) without altering coating formation. Physicochemical characterization using Fourier transform infrared spectroscopy (FTIR), X-ray photoelectron spectroscopy (XPS), thermogravimetric analysis (TGA), scanning electron microscopy (SEM), high-resolution transmission electron microscopy (HRTEM), the Brunauer–Emmett–Teller (BET) method, and the diffraction of x-rays (XRD) confirmed successful HF loading into ZIF-8 with intact crystal structures. Antifouling tests showed HF@ZIF-8 enhanced antibacterial inhibition against *Staphylococcus aureus* (97.28%) and *Escherichia coli* (>97%) and suppressed *Chromobacterium violaceum* CV026 pigment synthesis at 0.25 mg/mL (sub-growth concentration). The reconstructed PG/PVP/PEI/HF@ZIF-8 coating achieved 72.47% corrosion inhibition via synergistic anodic protection and physical shielding. This work provides a novel green approach for surface antifouling and drag reduction, highlighting MOF-loaded QSIs as promising additives to enhance the antifouling performance of hydrogel coatings, anti-corrosion performance, and QSI performance for sustainable marine engineering applications.

## 1. Introduction

Marine biofouling, defined as the colonization of submerged surfaces (e.g., ship hulls, offshore infrastructure, and marine equipment) by microorganisms, algae, and invertebrates, leads to the formation of persistent biofilms [1]. This phenomenon imposes substantial economic and operational burdens on maritime industries, primarily through increased hydrodynamic drag (15–50% higher fuel consumption and elevated carbon emissions), accelerated material degradation, and elevated maintenance costs [2]. Notably, biofouling-induced corrosion, driven by acidic metabolites secreted by fouling organisms, exacerbates the deterioration of metallic substrates, further amplifying lifecycle expenses [3]. Since the global prohibition of tributyltin (TBT)-based antifouling coatings in 2008, copper-based alternatives have dominated ~80% of the commercial antifouling market. While less ecotoxic than TBT compounds, their environmental persistence remains contentious among regulatory agencies, casting uncertainty on their long-term viability [4]. Contemporary strategies, such as low-surface-energy coatings (e.g., silicone-based polymers and fluoropolymers), minimize bioadhesion through physicomechanical resistance. However, their efficacy is intrinsically linked to hydrodynamic shear forces, rendering them suboptimal for static structures (e.g., port-harbored vessels and offshore platforms) [5,6]. Consequently, research prioritizes the development of environmentally benign alternatives capable of dual-phase antifouling action under both stationary and dynamic conditions.

In marine fouling environments, quorum sensing accelerates the aggregation of fouling microbial communities and rapid biofilm growth, thereby exacerbating the fouling of ship hulls and underwater infrastructures. Disrupting QS pathways has thus emerged as a critical research direction in marine antifouling strategies in recent years. Quorum sensing inhibitors, small molecules or bioenzymes that block or interfere with QS signal transduction, have undergone intensive investigation in marine antifouling research. Recent studies have reported diverse QSIs through screening, synthesis, and antifouling performance evaluation, focusing on natural product discovery, chemical synthesis, and microcarrier-based sustained-release systems. Diterpenoid derivatives (knightine) were isolated from the metazoan Eunicea knighti and their antifouling properties were validated via QS inhibition assays and biofilm suppression experiments [7]. The diterpenoids outperformed kojic acid and Cu_2_O in inhibiting biofilm formation. Ten brominated alkaloids were screened from secondary metabolites of the North Sea bryozoan Flustra foliacea, with most compounds significantly disrupting early-stage biofilm formation by marine bacterial strains [8]. Additionally, multiple marine-derived QSIs were identified using the purple bacterium CV017, showing that kojic acid and oxo-pyrone effectively inhibited bacterial and Amphora coffeaeformis attachment [9]. These studies highlight the rich reservoir of QSI molecules in marine secondary metabolites with antifouling potential. The effects of QSIs on biofilms of Pseudoalteromonas and sulfate-reducing bacteria in marine corrosion product layers were investigated using D-amino acids and QS signaling molecules as models [10,11]. Through biofilm quantification and microscopic structure analysis, QSIs were demonstrated to effectively disrupt QS pathways and suppress biofilm formation. Ca^2+^-bound poly(lactic-co-glycolic acid) (PLGA) microparticles were designed to maintain sufficient QSI concentration on hydroxyapatite (HA) surfaces, effectively preventing biofilm formation on HA substrates [12]. This work provides a novel framework for sustained QSI release and long-acting antifouling applications. While these studies underscore the efficacy of QSIs in inhibiting marine fouling biofilms and offer theoretical and technical support for developing advanced antifouling systems, practical applications of QSIs are constrained by challenges such as poor stability, susceptibility to degradation, and short release cycles, which limit their long-term antifouling performance in marine environments.

Inspired by natural hydrogels secreted by aquatic organisms (primarily composed of bioadhesive mucins) to resist fouling organism attachment, synthetic hydrogels have emerged as promising candidates in marine antifouling research. The unique microporous architecture and diverse chemical crosslinking of hydrogels enable functional modification, facilitating their compatibility with medical, mechanical, and electrical applications [13]. A hydrogel coating was developed using poly(sulfobetaine methacrylate) (PSBMA) as the matrix and gentamicin sulfate (GS) as the biocide, demonstrating dual capabilities against macromolecular adhesion and bacterial infection in vitro and in vivo [14]. Hydrophilic antifouling hydrogel surfaces swell upon water absorption, forming a hydrated layer that acts as a physical barrier to block the initial attachment of proteins and polysaccharides in seawater—the first stage of marine fouling [13]. A self-cleaning, oil-resistant, and regenerable hydrogel coating was fabricated via the physical mixing of sodium polyacrylate (PAAS) and silicone resin, with field tests in marine environments confirming its superior antifouling performance [15]. Challenges of weak adhesion and uneven coverage in the large-scale application of hydrophilic polymers were addressed by spray-coating a hydrogel composed of four-armed polyethylene glycol and acrylamide onto an epoxy resin interlayer, a system that exhibited excellent self-cleaning properties while maintaining robust adhesion in marine conditions [16]. A dual-component hydrogel with low swelling in saline solutions was created by combining the antipolyelectrolyte effect of PSBMA (poly-N-(3-sulfopropyl)-N-(methacryloxyethyl) N, N-dimethylammonium betaine) with the typical polyelectrolyte behavior of polyacrylic acid (PAA), a coating that not only resists fouling but also withstands abrasion from sand-laden water [17]. A PVA-based hydrogel incorporating spherical Cu_2_O–tannic acid (TA) submicron particles was developed via cyclic freezing–thawing, enabling the sustained release of copper ions for antifouling while maintaining robust mechanical properties [18]. Capsaicin-derived N-(4-hydroxy-3-methoxybenzyl)acrylamide (HMBA) was introduced into polyvinyl alcohol to form a double-network hydrogel with low swelling ratio, achieving stable capsaicin release and superior antifouling efficacy [19].

To enhance the stability and sustained-release performance of antifouling agents, metal-organic frameworks have gained extensive attention in antifouling applications due to their high specific surface area, tunable pore size, structural stability, and excellent drug-loading capacity [20]. Despite the instability of MOFs with open metal nodes in aqueous or humid environments, their potential in antifouling lies in the slow release of metal ions (e.g., Cu^2+^) to inhibit fouling organism attachment. For example, Cu_2_O@Cu-MOF sustained-release capsules embedded in acrylic resin coatings hydrolyze in seawater to gradually release Cu^2+^, with acid-sensitive Cu-MOF triggering enhanced ion release at fouling sites via microbial acidic metabolites [21]. Another study integrated Cu-MOF-74 and carboxylated multi-walled carbon nanotubes (MWCNT-COOH) into self-healing polymers, where Cu-MOF-74 enables stable Cu^2+^ release and MWCNT-COOH minimizes ion loss, yielding coatings with superior antibacterial performance [22]. Duan et al. [23] prepared ZIF-8 nanoparticles via a microemulsion method and directly incorporated them into epoxy resin to fabricate anti-corrosive coatings. Testing results showed that the anti-corrosive performance of the coatings improved with the increase in the mass fraction of ZIF-8 nanoparticles. The surface modification of MOFs further enhances coating antifouling efficiency by optimizing ion release kinetics and mechanical durability.

To fabricate a mussel-inspired hydrogel coating with quorum sensing inhibitor functionality, 2-(5H)-furanone (HF)-loaded ZIF-8 nanoparticles were incorporated into a polyglycerol/polyvinylpyrrolidone/polyethyleneimine (PG/PVP/PEI) hydrogel matrix. Physicochemical characterizations confirmed successful HF encapsulation within ZIF-8 and uniform dispersion in the hydrogel. Surface attachment assays using marine fouling organisms demonstrated the coating’s enhanced antibacterial and antifouling efficacy. Notably, HF@ZIF-8 exhibited potent QS inhibition, suppressing violacein production in *Chromobacterium violaceum* CV026 at sub-inhibitory concentrations (0.25 mg/mL). The reconstructed coating synergistically combined anodic protection and physical barrier effects, significantly improving corrosion inhibition efficiency. The coating biomimetically replicates mussel-adhesive molecular architecture, incorporating ZIF-8 MOFs loaded with QSI. This design unites physical barrier protection, QS, and corrosion suppression within a singular system. Unlike conventional coatings, our methodology replaces metal ion crosslinkers with functional MOFs, achieving simultaneous structural integrity and bioactive functionality. This cohesive strategy represents an eco-innovative solution for sustainable marine antifouling applications.

## 2. Results and Discussion

### 2.1. Characterization of ZIF-8 and HF@ZIF-8

**Figure 1:**
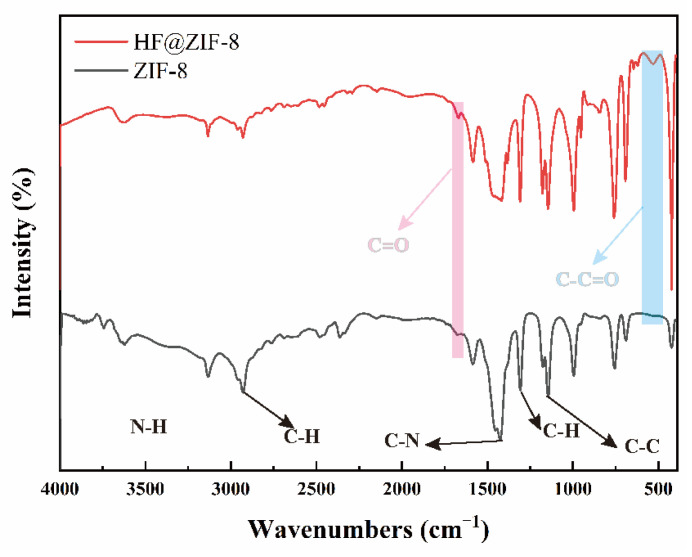
The synthesized ZIF−8 and HF@ZIF−8 FTIR spectrum.

FTIR was employed to compare the characteristic absorption peaks of ZIF-8 and HF@ZIF-8 powders before and after drug loading, verifying the successful encapsulation of HF in ZIF-8. Figure 1 shows the FTIR spectra of as-synthesized ZIF-8 and HF@ZIF-8 (with air as the background). The spectra exhibit high consistency before and after loading: peaks at 2930 cm^−1^ and 995 cm^−1^ originate from C-H stretching and out-of-plane bending vibrations of 2-methylimidazole, respectively; a medium-intensity peak at 3133 cm^−1^ corresponds to N-H group stretching vibrations; a doublet at 1427 cm^−1^ is attributed to C-N stretching of primary amide groups in the 2-methylimidazole ligands; a medium-intensity peak at 1145 cm^−1^ arises from C-C stretching in the imidazole heterocycle; and a strong absorption at 422 cm^−1^ confirms the formation of Zn-N bonds in ZIF-8 [24]. These peaks not only indicate the successful synthesis of the ZIF-8 metal-organic framework but also demonstrate that HF loading did not disrupt its physical structure. After HF loading, weak peaks at 1670 cm^−1^ (C=O stretching) and 532 cm^−1^ (C-C=O stretching) appear in the HF@ZIF-8 spectrum [25], originating from the molecular structure of HF, providing preliminary evidence of successful HF encapsulation in ZIF-8.

**Figure 2:**
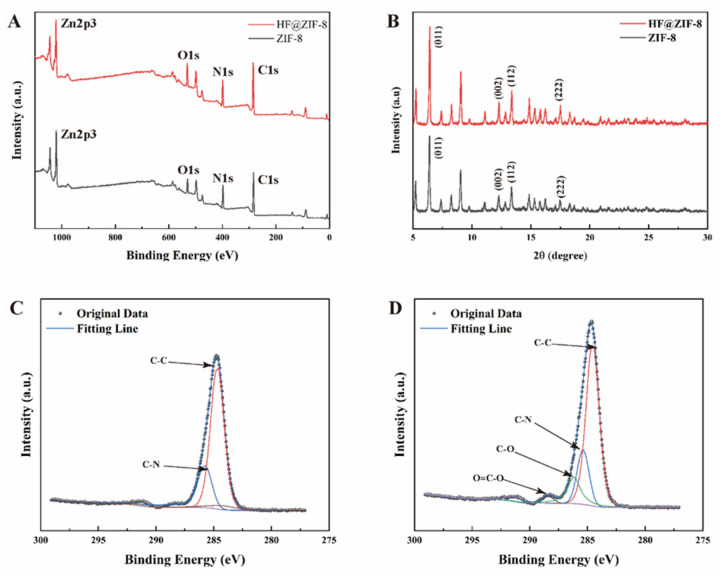
XPS and XRD spectra of ZIF-8 and HF@ZIF-8. (**A**) ZIF-8 and HF@ZIF-8 XPS full spectrum; (**B**) ZIF-8 and HF@ZIF-8 XRD pattern; (**C**) C1s fine spectrum of ZIF-8; and (**D**) HF@ZIF-8 C1s fine spectrum.

An analysis of the synthesized samples by FTIR initially indicated that the successful loading of HF into ZIF-8 did not alter the basic physical properties of ZIF−8, though the FTIR spectra showed no obvious differences. Therefore, XPS and XRD were used for further analysis of the samples, with results shown in Figure 2. The elemental contents analyzed by full-spectrum scanning are listed in Table 1, where the proportion of O element increased from 9.03 at% to 11.72%, originating from the C=O and C-C=O functional groups in HF. The C1s fine spectra fitting of ZIF-8 and HF@ZIF-8 is shown in Figure 2C,D. The as-synthesized pristine ZIF-8 only contained C-C at 284.73 eV and C-N at 285.78 eV, which are attributed to 2-methylimidazole [26]. After HF loading, additional peaks appeared in the C1s fine spectrum at 286 eV (C-O) and 288.5 eV (O=C-O), which can only originate from HF. These results are highly consistent with the FTIR spectra, further confirming the successful loading of HF into ZIF-8. The XRD patterns of ZIF-8 before and after HF loading are shown in Figure 2B, both exhibiting characteristic diffraction peaks of ZIF-8. High-resolution diffraction peaks at 2*θ* = 6.42°, 12.29°, 13.36°, and 17.52° correspond to the (011), (002), (112), and (222) crystal planes of ZIF-8 [27], respectively. This indicates that both the synthesized ZIF-8 and HF-loaded HF@ZIF-8 are pure-phase ZIF-8 materials, and the limited loading of HF on ZIF-8 allows the loaded ZIF-8 to maintain a good crystalline structure. Unlike hydrogels where metal ions directly participate in coordination crosslinking, the zinc ions in ZIF-8 remain structurally confined within the metal-organic framework without engaging in ionic crosslinking. This configuration stabilizes the hydrogel network primarily through hydrogen-bonding and electrostatic associations.

**Figure 3:**
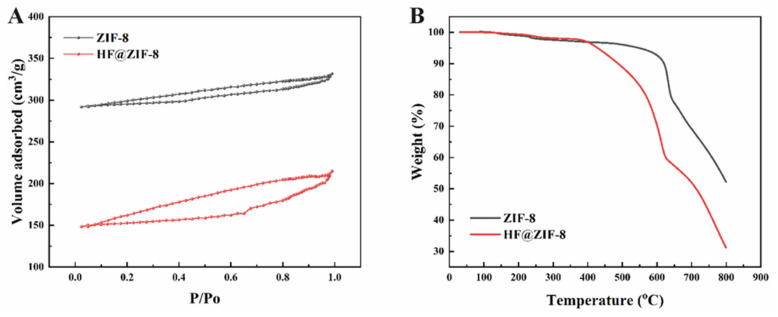
Adsorption and desorption BET curve of each sample (**A**). Thermogravimetric analysis curve of each sample (**B**).

Figure 3A displays the nitrogen adsorption–desorption isotherms of ZIF-8 samples before and after HF loading. Both samples exhibit Type I isotherms, indicating an abundance of microporous structures on their surfaces. As listed in Table 2, BET surface area analysis and Barrett–Joyner–Halenda (BJH) pore size analysis show that the specific surface area of the as-prepared ZIF-8 is 1157.483 m^2^/g, which decreases sharply to 613.519 m^2^/g after HF loading. Concurrently, the average pore diameter decreases from 3.699 nm to 2.971 nm, and the pore volume reduces from 0.099 cm^3^/g to 0.061 cm^3^/g. This phenomenon is attributed to HF occupying the microporous structures of ZIF-8, forming mesoporous structures through HF encapsulation and increasing the size of metal-organic ligand interactions, further validating the effectiveness of HF@ZIF-8 loading [28]. TGA curves of ZIF-8 and HF@ZIF-8 with increasing temperature are shown in Figure 3B. Both materials exhibit negligible mass loss below 400 °C, demonstrating sufficient thermal stability for applications in marine antifouling coatings. After 400 °C, significant mass loss begins for both materials, with HF@ZIF-8 showing a notably faster mass decline rate than ZIF-8. This is due to the rapid decomposition and volatilization of internally loaded HF in HF@ZIF-8, coupled with the collapse of ZIF-8 particle structures upon heating. After 600 °C, the loaded HF and organic ligands in both ZIF-8 and HF@ZIF-8 are completely removed, with mass loss rates tending to be linear and constant.

**Figure 4:**
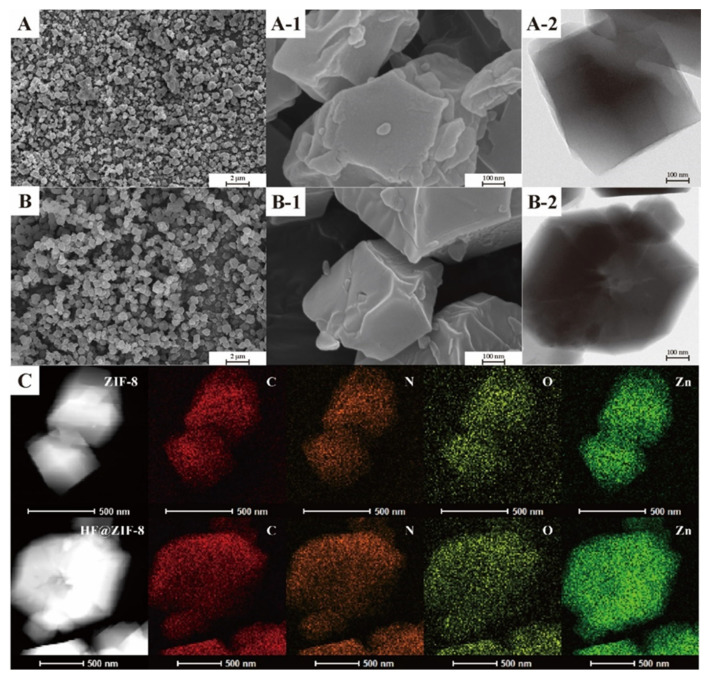
SEM and HRTEM images of each sample. (**A**) SEM images of ZIF-8. (**B**) SEM images of HF@ZIF-8. (**C**) HRTEM mapping element distribution maps for ZIF-8 and HF@ZIF-8. (-1 is a further enlarged image of the corresponding SEM images, and -2 are the HRTEM images of the corresponding sample.)

SEM and HRTEM were employed to characterize the surface morphology of ZIF-8 before and after HF loading. SEM images (Figure 4A,A-1) reveal that the synthesized ZIF-8 exhibits an average particle size of ~500 nm under a Zn^2+^/organic ligand molar ratio of 1:8. After HF loading, the average particle size of HF@ZIF-8 increases to ~700 nm (Figure 4B,B-1). Prior to loading, ZIF-8 displays distinct two-dimensional lamellar structures on its surface, whereas HF@ZIF-8 presents a smoother, less wrinkled surface morphology, indicating the effective encapsulation of ZIF-8 by HF. HRTEM images (Figure 4A-2,B-2) show that HF@ZIF-8 exhibits darker contrast under electron beam irradiation compared to pristine ZIF-8, attributed to the filling and coating of ZIF-8’s surface and pore structures by HF. Elemental mapping via TEM (Figure 4C) confirms the uniform distribution of elements in HF@ZIF-8, further demonstrating that HF loading does not alter the fundamental physical properties of ZIF-8.

### 2.2. Quorum Sensing Inhibition Performance of ZIF-8 and HF@ZIF-8

**Figure 5:**
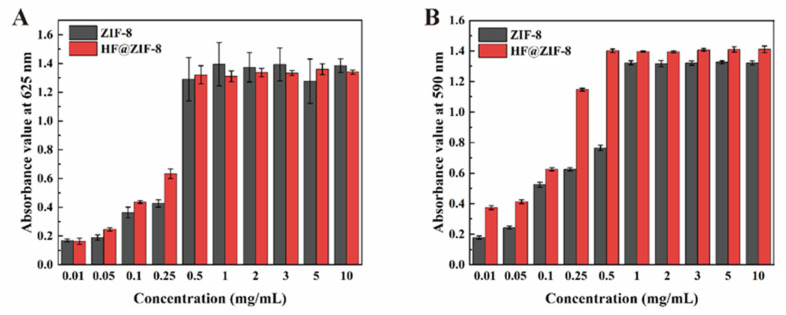
The absorbance of CV026 suspension at different concentrations of ZIF-8 and HF@ZIF-8. (**A**) OD 625 nm. (**B**) OD 590 nm.

The QSI effects of ZIF-8 before and after HF loading were evaluated using *Chromobacterium violaceum* CV026 following the method reported by Martin et al. [29]. *C. violaceum* CV026, a Gram-negative bacterium, serves as a screening model for QS inhibition (QSI) activity, as its violacein production is tightly regulated by the quorum sensing system [30]. Wild-type *C. violaceum* can endogenously produce signaling molecules to regulate violacein synthesis, whereas CV026, a mini-Tn5 mutant, lacks the ability to generate endogenous signals and thus does not produce violacein unless exogenous signaling molecules are added [31]. In this study, N-butyryl-L-homoserine lactone (C4-HSL) was used as the exogenous signal molecule to induce violacein production in CV026. The QS inhibition performance of HF@ZIF-8 was characterized by quantifying violacein production via OD_590_ measurements of ethanol-dissolved samples, as shown in Figure 5B. A parallel control group without C4-HSL was set up to measure OD_625_ values at the same concentrations, quantifying the growth inhibition of CV026 by ZIF-8 and HF@ZIF-8 (Figure 5A). Comparative results showed that in the AHL-negative group, the growth inhibition of CV026 by both ZIF-8 and HF@ZIF-8 increased with concentration, peaking at 0.5 mg/mL, indicating that HF loading had no significant effect on growth inhibition. In the AHL-positive group, HF@ZIF-8 exhibited a stronger inhibition of violacein synthesis than the unloaded ZIF-8 at concentrations of 0.01–0.5 mg/mL, with a significant inhibition of purple pigment production observed even at a sub-inhibitory concentration of 0.25 mg/mL. These results demonstrate that HF-loaded ZIF-8 possesses quorum sensing inhibition capability.

### 2.3. Antifouling Performance

**Figure 6:**
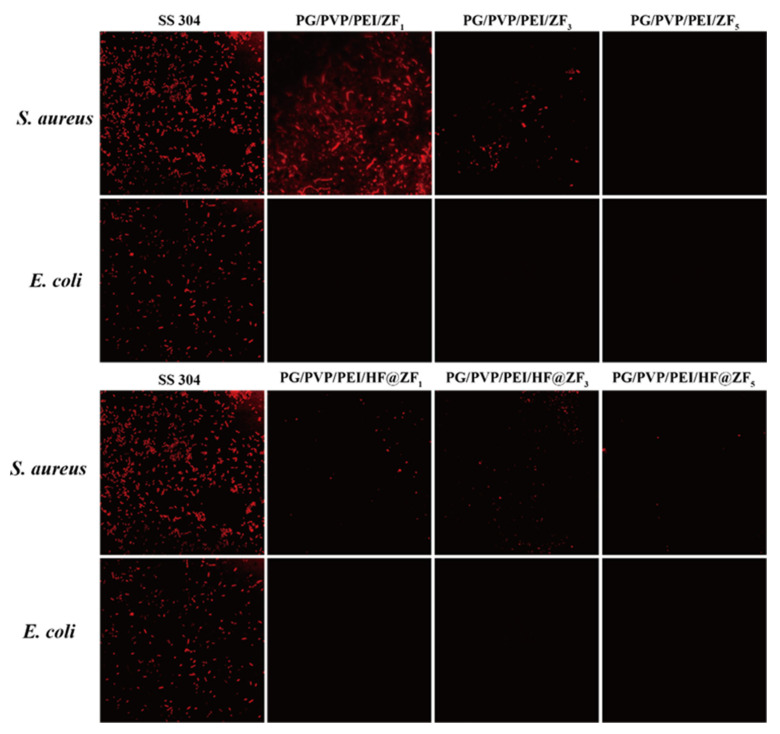
CLSM images of bacterial adhesion on different sample surfaces.

**Figure 7:**
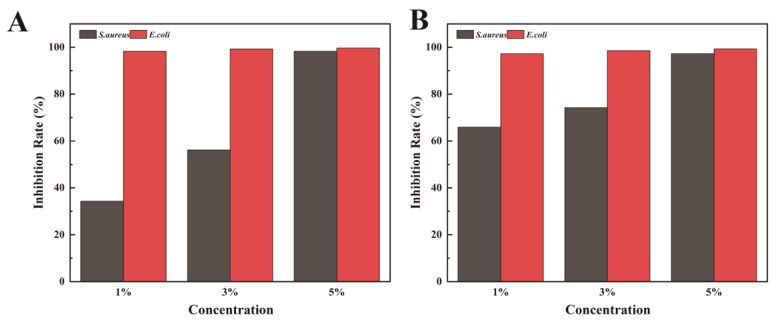
The inhibition rate of samples containing ZIF-8 and HF@ZIF-8 (**A**,**B**).

The densely crosslinked architecture formed by PG-PEI confers reduced surface wettability, thereby elevating overall hydrophobicity for improved interfacial water barrier performance. The synergistic interplay between PVP and PG-PEI collaboratively optimizes coating adhesion, durability, and antifouling efficacy. Furthermore, PG exhibits potent intrinsic antimicrobial properties, inducing growth suppression in marine fouling organisms at ultralow concentrations—a supplementary mechanism contributing to the coating’s antifouling performance [32]. The samples co-incubated with *S. aureus* and *E. coli* were fixed, stained, and then observed by confocal laser scanning microscopy (CLSM), with bacterial adhesion on the sample surfaces shown in Figure 6. The fluorescence intensity was quantified using ImageJ software v1.5.4, and the bacteriostatic rates calculated by Equation (1) are presented in Figure 7. Results indicate that when the ZIF-8 concentration in the hydrogel prepolymer was 1%, the coating exhibited weak inhibition against *S. aureus*, with a bacteriostatic rate of only 34.28%. When the concentration increased to 3%, the inhibition efficiency significantly rose to 56.14%, and further increasing the concentration to 5% resulted in a bacteriostatic rate of 98.25% (Figure 7A). For HF@ZIF-8, at a concentration of 1% in the prepolymer, the bacteriostatic rate against *S. aureus* was 65.89%, which increased to 74.23% at 3% and reached 97.28% at 5%. For *E. coli*, the bacteriostatic rates of coatings with 1%, 3%, and 5% ZIF-8 or HF@ZIF-8 in the prepolymer all exceeded 97%. This is attributed to the broad-spectrum bactericidal effect of zinc ions, while the QSI-loaded coatings slowly release HF with bacteriostatic activity, further enhancing the inhibitory effect against *S. aureus* when the hydrogel prepolymer contains the same concentration of HF@ZIF-8. The ZIF-8 component introduces a porous architecture that dramatically amplifies surface area, enabling both an efficient payload accommodation of quorum sensing inhibitors and their sustained release kinetics. The resultant hybrid system establishes a multifunctional barrier integrating physical occlusion with bioactive functionality, ultimately achieving effective antifouling performance.

### 2.4. Corrosion Inhibition Analysis

**Figure 8:**
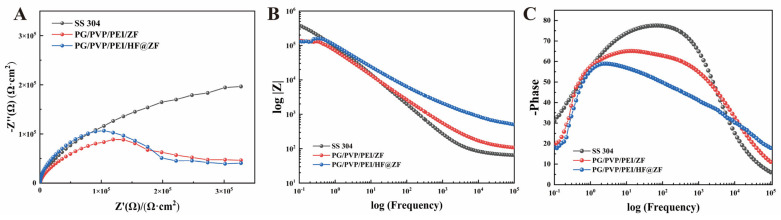
EIS experimental results. (**A**) Nyquist curves of each sample electrode. (**B**) Bode-modulus plot. (**C**) Bode-phase diagram.

The electrochemical corrosion performance of 304 SS coated with hydrogel was analyzed by measuring electrochemical impedance spectroscopy (EIS), as shown in Figure 8. The radius of the impedance arc is positively correlated with corrosion protection performance, where a larger arc indicates higher charge transfer resistance on the metal substrate surface. The Nyquist plots in Figure 8A show that spraying PG/PVP/PEI/ZF coating reduces the impedance arc, suggesting weak coating compactness and poor electrolyte isolation. In contrast, spraying PG/PVP/PEI/HF@ZF results in a larger impedance arc than the pristine 304 SS surface, attributed to the abundant C=O active groups in HF that form hydrogen bonds with PG, PVP, and PEI, thereby enhancing the coating’s water barrier properties. The Bode magnitude and phase angle plots of each sample are shown in Figure 8B,C. For Bode plots, the impedance modulus at low frequency (|Z|f = 0.01) and the phase angle at high frequency are critical indicators for evaluating corrosion protection. The pristine 304 SS exhibits a low-frequency impedance modulus of 3.82 × 10^5^ Ω, while coating with PG/PVP/PEI/ZF and PG/PVP/PEI/HF@ZF reduces the modulus to 1.40 × 10^5^ Ω and 1.30 × 10^5^ Ω, respectively. Compared to PG/PVP/PEI/ZF, the PG/PVP/PEI/HF@ZF coating shows an increasing trend in impedance modulus at 0.25 Hz, reaching 1.54 × 10^5^ Ω, higher than 1.26 × 10^5^ Ω of PG/PVP/PEI/ZF at the same frequency. Additionally, above 0.4 Hz, the impedance modulus of PG/PVP/PEI/HF@ZF exceeds that of 304 SS, demonstrating superior corrosion protection performance. The anti-corrosion mechanism stems from synergistic physicochemical effects within the coating architecture. ZIF-8 particles dispersed throughout the hydrogel matrix reinforce barrier efficacy by enhancing coating density and suppressing the permeation of corrosive species [33]. Simultaneously, ZIF-8 carriers controlling QSI release generate zinc-enriched microdomains that facilitate protective oxide layer formation on metal substrates through anodic passivation [34].

**Figure 9:**
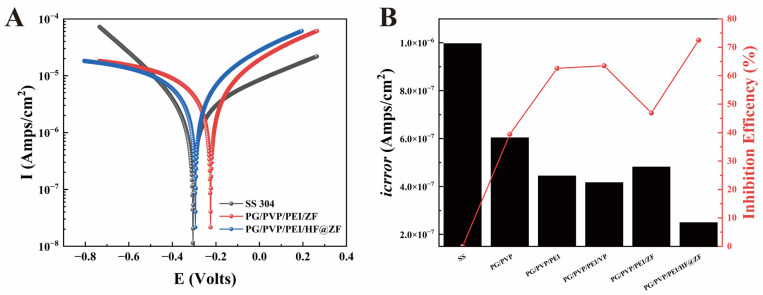
Tafel curves (**A**) for each electrode with corrosion parameters and corrosion inhibition rates obtained from the fitted curves (**B**).

Further corrosion resistance of the samples was evaluated via Tafel polarization curve measurements, with fitting results shown in Figure 9A. The corrosion potential (*E_corr_*) and corrosion current density (*i_corr_*) derived from the fitting are listed in Table 3. Using icorr in Equation (2), the corrosion inhibition rates of the coatings were calculated and presented in Figure 9B. For the 304 SS, *E_corr_* was −0.305 V, and decreased to −0.478 V after coating with PG/PVP/PEI/ZF, whereas coating with PG/PVP/PEI/HF@ZF increased *E_corr_* to −0.294 V, indicating enhanced anti-corrosion capability consistent with the trends observed in Nyquist plots. The *i_corr_* of the 304SS substrate was 0.908 μA·cm^−2^, decreasing to 0.482 μA·cm^−2^ for the PG/PVP/PEI/ZF coating (46.92% inhibition rate). The PG/PVP/PEI/HF@ZF coating exhibited a significantly lower *i_corr_* of 0.250 μA·cm^−2^ and a higher inhibition rate of 72.47%, outperforming the PG/PVP/PEI/V_2_O_5_ coating (with V_2_O_5_) in corrosion protection efficiency (Table 4). This improvement is attributed to the formation of a zinc-rich coating upon replacing VP with ZIF-8, which provides anodic protection, and the crosslinking of active C=O moieties in HF with the hydrogel matrix, enhancing coating compactness and physical shielding effects [34]. Concurrently, the PG-PEI network introduces robust covalent and coordination crosslinks, strengthening structural resilience to augment corrosion resistance [35]. Benchmarked against prior developments, this investigation achieves an unprecedented trifunctional integration of antifouling, corrosion inhibition, and quorum quenching, demonstrating superior multifunctional performance. A subsequent comparative analysis examines electrochemical protection and biofouling resistance metrics relative to representative ZIF-based coatings in contemporary literature (Table 4).

## 3. Conclusions

The ZIF-8 metal-organic framework loaded with HF was doped into the PG/PVP/PEI hydrogel coating. A physicochemical characterization of ZIF-8 before and after HF loading was conducted to confirm successful HF encapsulation. Using 304 stainless steel as the substrate, antifouling and anti-corrosion tests of the hydrogel coating were performed, yielding the following conclusions:

(1) FTIR, XPS, and TGA analyses confirmed the successful loading of HF into ZIF-8. SEM, HRTEM, BET, and XRD results indicated the effective encapsulation of ZIF-8 by HF, with no disruption to the crystalline structure of ZIF-8 before and after HF loading.
(2) Antifouling performance tests using *Staphylococcus aureus* and *Escherichia coli* showed that when the concentrations of ZIF-8 in the hydrogel prepolymer were 1%, 3%, and 5%, the inhibition rates against *S. aureus* were 34.28%, 56.14%, and 98.25%, respectively. Replacing ZIF-8 with HF@ZIF-8 increased the inhibition rates to 65.89%, 74.23%, and 97.28%, respectively. For *E. coli*, all samples exhibited inhibition rates exceeding 97%.
(3) QSI analysis of HF@ZIF-8 using *Chromobacterium violaceum* CV026 showed that the growth inhibition of CV026 by both ZIF-8 and HF@ZIF-8 peaked at a concentration of 0.5 mg/mL. At concentrations of 0.01–0.5 mg/mL, HF@ZIF-8 demonstrated a stronger inhibition of violacein synthesis in CV026 compared to unloaded ZIF-8, with a significant inhibition of purple pigment production observed even at a sub-inhibitory concentration of 0.25 mg/mL, confirming the quorum sensing inhibition capability of HF@ZIF-8.
(4) The PG/PVP/PEI/HF@ZF hydrogel coating exhibited a high corrosion inhibition rate of 72.47%, attributed to the synergistic effects of the anodic protection and physical barrier provided by the zinc-rich ZIF-8 and crosslinked HF within the hydrogel matrix.

## 4. Materials and Methods

### 4.1. Materials and Reagents

Chemical reagents: Propidium iodide (PI) and glutaraldehyde were obtained from Shanghai Aladdin Biochemical Technology Co., Ltd. (Shanghai, China); 2-methylimidazole, phloroglucinol (PG), quorum sensing inhibitor (HF), and branched polyethylenimine (PEI; MW 1800, 99% purity) were commercially sourced from Shanghai Macklin Biochemical Co., Ltd. (Shanghai, China). Additional compounds included polyvinylpyrrolidone (PVP K90) and anhydrous ethanol (>99.7% purity), which were purchased from Sinopharm Chemical Reagent Co., Ltd. (Shanghai, China). Culture media: Luria-Bertani (LB) broth and LB agar were obtained from Shanghai Bio-way technology Co., Ltd. (Shanghai, China); BG11 medium was supplied by Qingdao Hope Bio-technology Co., Ltd. (Shandong, China). All chemicals were analytical-grade reagents employed without additional purification.

### 4.2. ZIF-8 Synthesis and HF Loading

**Figure 10:**
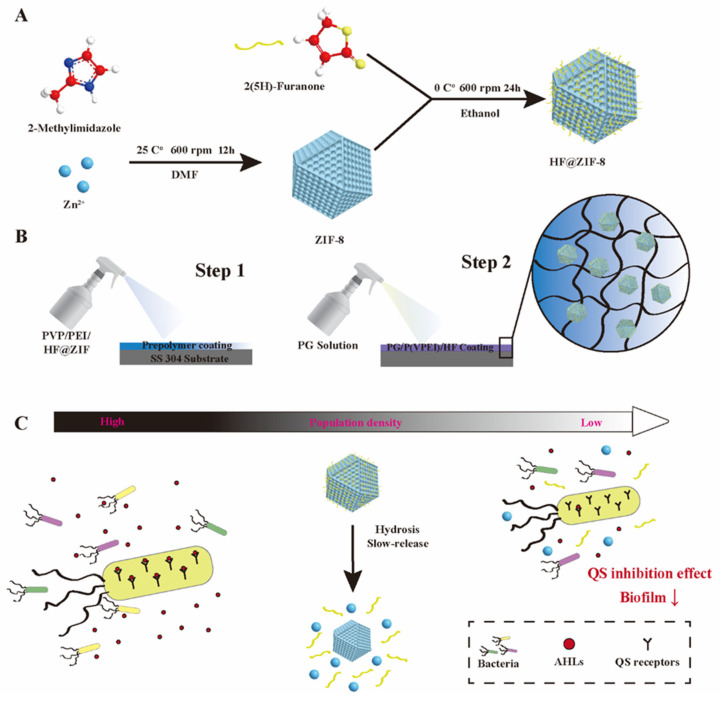
The main schematic diagram of (**A**) the synthesis of ZIF-8 and the loading process of HF. (**B**) Preparation process of modified ZIF-8 hydrogel coating. (**C**) Schematic diagram of QS inhibition effect.

As shown in Figure 10A, 9 g of Zn(NO_3_)_2_·6H_2_O and 19.704 g of 2-methylimidazole were dissolved in 250 mL of DMF under vigorous stirring (600 rpm) at room temperature for 12 h. The resulting white precipitate was collected via centrifugation, washed with anhydrous ethanol under ultrasonic treatment for 30 min, and freeze-dried for 12 h to obtain ZIF-8 with a metal-to-ligand molar ratio of 1:8, which exhibits optimal drug-loading capacity.

For HF loading, 2 g of ZIF-8 was dispersed in 200 mL of HF-ethanol solution (20 mg/mL) to achieve a ZIF-8:HF mass ratio of 1:2. The mixture was stirred in an ice-water bath for 24 h, followed by centrifugation and freeze-drying for 12 h to yield HF@ZIF-8.

### 4.3. Preparation of ZIF-8 and HF@ZIF-8 Composite Hydrogel Coatings

PVP/PEI prepolymer was prepared via the direct mixing of PVP and PEI solutions. In fabricating the hydrogel coating, PVP functions as a flexible hydrophilic matrix that enhances film formation and substrate adhesion via hydrogen-bonding interactions [40]. Specifically, PVP was heated at 80 °C with magnetic stirring at 800 rpm to prepare a 50 mg/mL solution, which was then degassed in a vacuum oven to eliminate air bubbles after complete dissolution. Meanwhile, 99 vol% PEI solution was diluted to 50 vol%, mixed with the pre-prepared PG solution at a volume ratio of 1:10, and transferred to a spray tank to obtain the PVP/PEI prepolymer. To prepare the PVP/PEI/ZIF-8 and PVP/PEI/HF@ZIF-8, the as-synthesized PVP/PEI prepolymer was first modified by incorporating 0.1 vol% sodium polycarboxylate dispersant under magnetic stirring at 600 rpm for 30 min to ensure homogeneous mixing. Subsequently, 1 wt% of ZIF-8 and HF@ZIF-8 powder were added slowly into the solution separately while maintaining continuous ultrasonic vibration (200 W, 40 kHz) for 2 h to achieve uniform dispersion. The ultrasonic treatment was carried out in an ice-water bath to prevent overheating and potential structural damage to ZIF-8 and HF@ZIF-8. The mixed pre-prepared PG solutions at a volume ratio of 1:10 were sprayed to obtain coatings (Figure 10B). The concentrations of ZIF-8 and HF@ZIF-8 in the prepolymers were adjusted to 1%, 3%, and 5% to optimize antifouling performance, resulting in coatings denoted as PG/PVP/PEI/ZF1, PG/PVP/PEI/ZF3, and PG/PVP/PEI/ZF5, and PG/PVP/PEI/HF@ZF1, PG/PVP/PEI/HF@ZF3, and PG/PVP/PEI/HF@ZF5.

### 4.4. Material Characterization

The surface morphology and internal structure of ZIF-8 before and after HF loading were observed using a Hitachi S-4800 field-emission scanning electron microscope (SEM, Hitachi, Ltd., Tokyo, Japan) and a HRTEM equipped with an energy filtering system (accelerating voltage: 200–300 kV). The SEM acquired particle morphology and dispersion state at an accelerating voltage of 5–15 kV and a working distance of 8–10 mm, while the HRTEM combined with EDS elemental mapping analyzed the distribution of F elements to confirm the loading position of HF. Fourier transform infrared spectroscopy (FT-IR) spectra were collected using an Agilent Cary 610/670 spectrometer (Agilent Technologies, Inc., Santa Clara, CA, USA) in the range of 4000–400 cm^−1^ with 128 scans at a resolution of 4 cm^−1^. The average of 64 scans was baseline-corrected and CO_2_ peaks were eliminated using Resolution Pro software version 5.4.1, followed by the comparison of imidazole ring (1580–1620 cm^−1^), Zn-N bond (420–450 cm^−1^), and HF characteristic peaks (e.g., C=O vibration at 1750–1780 cm^−1^) to verify functional group interactions. X-ray photoelectron spectroscopy (XPS) was performed using a Thermo Fisher ESCALAB 250Xi instrument (Thermo Fisher Scientific, Inc., Waltham, MA, USA) with monochromatic Al Kα radiation (1486.6 eV), scanning for Zn, N, C, O, and F elements and analyzing the chemical states of Zn 2p (1021–1023 eV), N 1s (398–402 eV), and F 1s (687–689 eV), with C 1s (284.8 eV) as the internal standard for charge correction. XRD was conducted using a Bruker D8 diffractometer (Bruker Corporation, Billerica, MA, USA) with Cu Kα radiation (λ = 1.5406 Å), scanning from 2θ = 5° to 50° at a rate of 0.02°/s, and comparing with the standard card (ZIF-8 PDF#01-089-3737) to confirm crystal structure integrity. Thermogravimetric analysis (TGA) was performed using a TA Instruments Q500 thermogravimeter (TA Instruments, Milford, Massachusetts, USA) under a nitrogen atmosphere (50 mL/min), heating from room temperature to 800 °C at 10 °C/min to calculate HF loading and evaluate thermal stability by comparing weight loss curves before and after loading. BET surface area and pore size analysis were conducted via nitrogen adsorption at 77 K after vacuum degassing the samples at 120 °C for 6–12 h, with specific surface area and pore size distribution calculated using the BET equation and DFT model to compare pore filling before and after loading. These characterization methods comprehensively verified the successful loading of HF in ZIF-8 and its physicochemical property changes from multiple dimensions, including morphology, functional groups, elemental valence states, crystal structure, thermal stability, and pore characteristics.

### 4.5. Quorum Sensing Inhibition Analysis of HF@ZIF-8

The quorum sensing (QS) inhibitory activity of HF@ZIF-8 was evaluated using *Chromobacterium violaceum* CV026, a reporter strain that produces violacein in response to exogenous acyl-homoserine lactone (AHL) signals. Two 36-well plates were prepared:

*AHL-negative plate*: To assess growth inhibition, CV026 cultures in kanamycin broth were treated with ZIF-8 or HF@ZIF-8 (0, 0.01, 0.05, 0.1, 0.25, 0.5, 1, 2, 3, 5, and 10 mg/mL) and incubated at 30 °C for 18 h. Optical density at 625 nm (OD_625_) was measured to quantify bacterial growth.

*AHL-positive plate*: To evaluate QS inhibition, CV026 cultures supplemented with 20 μg/mL C4-HSL were treated identically. After 18 h incubation, cultures were freeze-dried for 24 h and extracted with 1 mL of anhydrous ethanol (160 rpm, 6 h), and the OD_590_ was measured to quantify violacein production.

### 4.6. Antifouling Performance Evaluation

The antibacterial efficacy of the surfaces was evaluated against the model Gram-positive bacterium *Staphylococcus aureus* and Gram-negative bacterium *Escherichia coli*, both cultured in Luria-Bertani (LB) broth. For antibacterial assays, pristine and coated specimens were placed in 36-well plates, inoculated with 1 mL of bacterial suspension per well, and incubated at 37 °C for 24 h in a temperature-controlled incubator. Samples were subsequently rinsed with phosphate-buffered saline (PBS) to remove non-adherent bacteria and fixed in 2.5% glutaraldehyde solution at 4 °C for 6 h. Following fixation, specimens were stained with propidium iodide (PI, 50 μg/mL) in the dark. Bacterial adhesion morphology was visualized using a TCS SP8 STED confocal laser scanning microscope (CLSM; Leica Microsystems GmbH, Wetzlar, Germany). The adhesion ratio was quantified via ImageJ software and calculated using the established equation [41]:

(1) $$AR\%=\frac{A’}{A}\times 100\%$$

where *AR*% denotes the attachment rate, *A*′ the area of microbial attachment, and *A* the total area of the field of view.

### 4.7. Electrochemical Performance Evaluation

The coated working electrodes were fabricated by soldering a 0.5 mm^2^ copper wire onto the surface of a 10 × 10 × 1 mm SS 304 sheet using tin soldering. Residual rosin and flux were then removed via ultrasonic cleaning with absolute ethanol. After cleaning, the SS 304 substrate with the soldered copper wire was encapsulated in epoxy resin. Once the resin was fully cured, the surface was polished to expose a 10 cm^2^ working area. Coatings were applied to the working surface using the corresponding materials described in each section, followed by freeze-drying to obtain the coated working electrodes. Prior to experiments, the working electrodes were fully immersed in artificial seawater to swell, simulating the actual operational conditions of the coatings.

A three-electrode system was used for electrochemical corrosion performance evaluation, consisting of a 10 × 10 × 0.1 mm platinum sheet as the counter electrode and a saturated calomel electrode (SCE) as the reference electrode. Different samples were immersed in 3.5% NaCl solution, and electrochemical experiments were conducted using a Gamry Interface 1010E electrochemical workstation.

*Electrochemical impedance spectroscopy (EIS)*: EIS measurements were performed over a frequency range of 10 mHz to 100 kHz with a sinusoidal voltage perturbation of 10 mV. The EIS data were analyzed using ZView2 software version 5.4.1 to fit the equivalent circuit.

*Potentiodynamic polarization curves*: Polarization curves were obtained by scanning from −0.5 to 0.5 V (vs. SCE) at a rate of 5 mV/s at room temperature. The corrosion potential (*E_corr_*) and corrosion current density (*i_corr_*) were derived by fitting and analyzing the polarization curves using Gamry Echem Analyst software version 6.33.

*Electrochemical corrosion inhibition efficiency (IE)*: The IE of the hydrogel-coated SS 304 specimens was calculated using Equation (2):

(2) $$IE\%=\frac{i_{o}-i_{s}}{i_{o}}\times 100\%$$

where *i_o_* and *i_s_* are the corrosion current densities of the pristine 304 SS and coated samples, respectively, determined by Tafel polarization curve fitting.

## Figures and Tables

**Table 1:** XPS quantitative analysis results.

Sample Name	C (at%)	N (at%)	O (at%)	Zn (at%)
ZIF-8	20.75	12.22	9.03	58.0
HF@ZIF-8	20.95	11.47	11.72	55.86

**Table 2:** Specific surface area parameters of ZIF-8 and HF@ZIF-8.

Sample Name	Specific Surface Area (m^2^/g)	Average Pore Diameter (nm)	Pore Volume (cm^3^/g)
ZIF-8	1157.483	3.699	0.099
HF@ZIF-8	613.519	2.971	0.061

**Table 3:** Calculation of the resulting corrosion inhibition rate.

Sample Name	*E_corr_*/V	*i_corr_*/μA^.^cm^−2^	*IE%*
SS	−0.305	0.908	/
PG/PVP/PEI/ZF	−0.478	0.482	46.92%
PG/PVP/PEI/HF@ZF	−0.294	0.250	72.47%

**Table 4:** Comparative analysis against extant literature. N/A: not available.

Coating System	(*IE%*)	Antibacterial/Antifouling	Functions	Ref.
PG/PVP/PEI/HF@ZIF-8	72.47%	*E. coli*, *S. aureus* > 97%, *C. violaceum* QSI positive	Anti-corrosion, antibacterial, and QSI	This work
PG/PVP/PEI/VP	63.49%	*S. aureus* > 99%, *E. coli* > 87%	Anti-corrosion and antibacterial	[36]
In situ ZIF-8 nanosheets	>95%, 87.8% after 168 h	N/A	Anti-corrosion	[37]
ZnO@ZIF-8-SA conversion	>99.99%, 95.29% after 14 d	N/A	Superhydrophobic, slow-release, and anti-corrosion	[38]
ZIF-8 double- layer coating	Significant improvement in *E_corr_*	N/A	Surface inhibits adhesion	[39]

## Data Availability

The original contributions presented in this study are included in the article. Further inquiries can be directed to the corresponding authors.
